# Epidemiological characteristics and transmission dynamics of dengue in Ningbo City, China before, during, and after the COVID-19 pandemic interventions

**DOI:** 10.1371/journal.pntd.0014813

**Published:** 2026-10-30

**Authors:** Yanru Chu, Yunkang Zhao, Buasiyamu Abudunaibi, Yao Wang, Yanwu Zhang, Song Lei, Yang Mao, Tianfeng He, Tianmu Chen, Yi Chen

**Affiliations:** 1 Ningbo Municipal Center for Disease Control and Prevention, Ningbo, Zhejiang, People’s Republic of China; 2 State Key Laboratory of Vaccines for Infectious Diseases, Xiang An Biomedicine Laboratory, School of Public Health, Xiamen University, Xiamen, China; 3 School of Public Health, Chengdu Medical College, Chengdu, Sichuan, China; Monash University, AUSTRALIA

## Abstract

**Background:**

Ningbo experienced a local dengue outbreak in 2018–2019, followed by the implementation and relaxation of COVID-19 pandemic interventions. Evidence has been limited on dengue epidemiology and the transmissibility across these periods.

**Methods:**

We analyzed routine dengue case and mosquito surveillance data from 2017 to 2024, using descriptive analyses to assess the epidemiological characteristics, and a mathematical model was developed to estimate the basic reproduction number (*R*_*0*_).

**Results:**

A total of 266 dengue cases were reported, with no deaths. Before the pandemic interventions, 51.19% of cases were overseas-imported; no cases were reported during pandemic interventions, whereas 73.47% were domestically imported after pandemic interventions. The median age of cases was 38.50 years (IQR = 31.25-54.00), and 57.52% were unemployed, retired, or in commercial services. The onset-to-diagnosis interval shortened post-interventions (4.46 days vs. 2.67 days). Viral typing increased post-interventions (34.59% vs. 63.27%), with 83.70% of cases identified as dengue virus-1 infections. Dengue cases were reported across all districts, with higher proportions in Haishu (27.07%) and Yinzhou (24.44%). The incidence was 2.82 per 100,000 persons. Incidence did not differ significantly by sex, but was significantly higher among individuals 15–59 years and those aged ≥60 years than among those aged ≤14 years (3.18 and 3.01 per 100,000 persons, respectively, vs. 0.45 per 100,000 persons). Incidence was highest in Haishu and Jiangbei (6.90 and 4.93 per 100,000 persons, respectively). A post-intervention incidence rebound was observed, although no statistically significant differences were observed in the ≤ 14 age group, Beilun District, Ninghai County and Xiangshan County. *Aedes albopictus* was the only vector species. The mean Breteau Index was 16.07 (SD: 8.04) and decreased across phases, and rebounded in Jiangbei, Zhenhai, and Haishu districts. The mean BI was higher in summer compared to other seasons (*p* < 0.05). Model fit was strong (*R*^*2*^ > 0.95). The median *R*_*0*_ was higher in the outbreak years (3.46, IQR = 2.98-3.95) than years without local transmission (0.00, IQR = 0.00-0.00; *p* < 0.001). Median *R*_*0*_ was 0.00 (IQR = 0.00-0.71) over 2017–2024 due to the absence of local cases in most years.

**Conclusions:**

Dengue remains non-endemic in Ningbo, and is driven primarily by imported infections rather than sustained local transmission. Despite a reduction in diagnostic delay and vector density, post-PHSM incidence rebounded, and district-level heterogeneity indicates ongoing outbreak potential. Sustained surveillance, rapid diagnosis and district-tailored vector control are critical to prevent re-establishment of local transmission.

## Author summary

We examined how dengue changed in Ningbo, a coastal Chinese city where the disease is not usually established. We compared the years before, during, and after the public health measures used to control the coronavirus pandemic. We reviewed health records and mosquito surveys to understand who became ill, where cases occurred, and how mosquito numbers changed. We also used a computer-based model to assess the potential for dengue to spread locally. Among 266 reported cases, no cases occurred during the strictest control period. After the measures were relaxed, dengue returned, mainly in people infected elsewhere in China. Local spread occurred during outbreaks in 2018 and 2019, but it did not continue from year to year. Cases and mosquito numbers varied considerably between districts and were generally higher during the summer. Although people were diagnosed more quickly and mosquito numbers decreased overall, dengue incidence increased again in some districts after the control measures ended. These findings show that a city can remain at risk even when dengue is not permanently established. For Ningbo and other port cities, prevention should combine rapid diagnosis, attention to travel-related infections, continued mosquito monitoring, and targeted control in higher-risk districts.

## Introduction

Dengue is a vector-borne disease caused by dengue virus (DENV), which comprises four serotypes (DENV-1 to DENV-4). It is one of the major neglected tropical diseases and remains highly prevalent in tropical and subtropical regions. Approximately 50% of the global population is currently at risk, with an estimated 100–400 million infections annually [1]. The global dengue burden, particularly in East Asia, has increased substantially over recent decades and continues to pose a major public health challenge [2,3]. After a temporary decrease during 2020–2022, likely related to the COVID-19 pandemic interventions and under-reporting, dengue has resurged globally in 2023–2024 [4].

In China, dengue is classified as a class B notifiable infectious disease. All dengue cases must be reported to the China National Notifiable Infectious Disease Reporting Information System (NNDRS) within 24 hours [5]. Recurrent dengue epidemics over the past two decades have become an important public health concern [6,7]. Ningbo is a major coastal city with extensive domestic and international mobility and one of the world’s largest port systems [8]. Although historically considered non-endemic, with no local dengue cases before 2014, Ningbo experienced multiple local outbreaks between 2015 and 2018 [9].

Given these conditions, understanding the epidemiological characteristics and transmission dynamics of dengue in Ningbo is essential for early warning and targeted prevention. However, evidence describing the situation remains limited since 2018. To address this gap, we conducted a comprehensive analysis using descriptive epidemiology and mathematical modeling across three periods: before, during, and after the COVID-19 pandemic interventions (2017–2024).

## Methods

### Ethics statement

#### Ethics approval and consent to participate.

Data for this study were obtained from the Ningbo CDC. The study was exempt from ethical review, as the Medical Ethics Committee of the Ningbo CDC waived the requirement for informed consent for the following reasons: (1) the disease surveillance activity was part of routine public health surveillance; (2) all analysed data were anonymised; (3) no medical interventions or biological samples were involved; and (4) the study procedures and results did not affect patient clinical management.

### Study setting and design

Ningbo City (120°55′E to 122°16′E, 28°51′N to 30°33′N), located on the eastern coast of China, had a population of 9.78 million in 2024 (Fig 1A) [10]. It is administratively composed of six districts (Haishu, Jiangbei, Zhenhai, Beilun, Yinzhou, and Fenghua), two counties (Ninghai and Xiangshan), and two county-level cities (Cixi and Yuyao) (Fig 1A).

**Fig 1:**
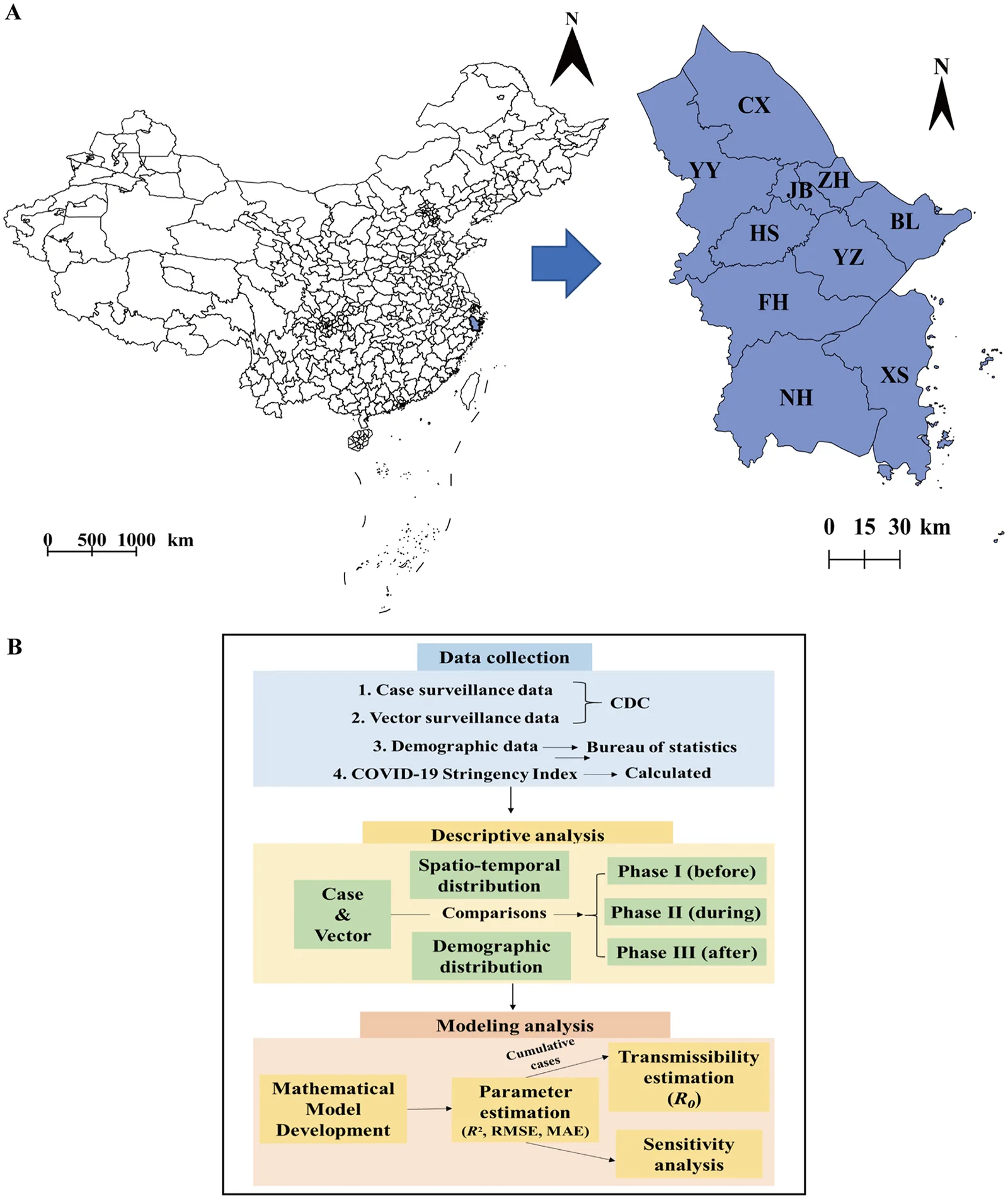
Study Setting and Design Overview. **(A)** The geographic location of Ningbo City. The left panel shows the geographic location of Ningbo within China, while the right panel presents a map of Ningbo’s administrative divisions. The base map uses national and citywide administrative boundaries from the Database of Global Administrative Areas (GADM, https://gadm.org/data.html; license: CC BY 4.0, https://gadm.org/license.html). CX, Cixi; YY, Yuyao; JB, Jiangbei; ZH, Zhenhai; HS, Haishu; BL, Beilun; YZ, Yinzhou; FH, Fenghua; XS, Xiangshan; NH, Ninghai. **(B)** Flowchart of the Study Design. R^2^, Coefficient of Determination; RMSE, Root Mean Square Error; MAE, Mean Absolute Error; *R*_*0*_, Basic Reproduction Number.

This study included two integrated components. First, we conducted a descriptive analysis of epidemiological characteristics of dengue, including local and imported cases and vector surveillance. Second, we developed a mathematical model to quantify dengue transmissibility. The study period was divided into three phases: Phase I (pre-COVID-19 pandemic interventions, January 1, 2017 to January 13, 2020), Phase II (during COVID-19 pandemic interventions, January 14, 2020 to January 7, 2023), and Phase III (post-COVID-19 pandemic interventions, January 8, 2023 to December 31, 2024). This phase definition followed local policy timelines from previous investigations [11,12] (Fig 1B).

All dengue and mosquito surveillance data were derived from routine public health activities conducted by the Ningbo Center for Disease Control and Prevention (Ningbo CDC). Because the dataset was anonymized and de-identified, institutional review and informed consent were not required.

### Data collection

Daily dengue surveillance data from Ningbo between 2017 and 2024, including age, sex, occupation, address, date of onset, and diagnosis, were obtained from the NNDRS. Surveillance definitions and case classification criteria are provided in Supplementary Text 1 in S1 Text. The analysis included clinically diagnosed and laboratory-confirmed cases, while suspected cases were excluded.

Monthly vector surveillance data for the same period, covering survey dates, surveillance areas, surveyed households, and detection rates of mosquitoes, were obtained from the Ningbo CDC. The Breteau Index (BI) was calculated to assess vector density (see Supplementary Text 2 in S2 Text for details).

Annual demographic data for Ningbo City for the same period were collected from the Ningbo City Statistical Yearbook released by the Ningbo Bureau of Statistics [10]. Additionally, the daily COVID-19 Stringency Index for the same period in Ningbo City was calculated to quantify the Public Health and Social Measures (PHSM) for COVID-19 restrictions [13].

### Mathematical model

We developed a mathematical model to estimate dengue transmissibility based on previous studies [9,14] (Fig 2). Briefly, the human population ($N_{p}$) is divided into imported infected individuals ($I_{i})$, susceptible individuals ($S_{p}$), exposed individuals $(E_{p}$), infectious individuals $(I_{p}$), asymptomatic individuals $(A_{p}$), and recovered/removed individuals $(R_{p})$. The mosquito population ($N_{m}$) is divided into susceptible mosquitoes ($S_{m}$*)*, exposed mosquitoes ($E_{m}$), and infectious mosquitoes ($I_{m}$*)*.

**Fig 2:**
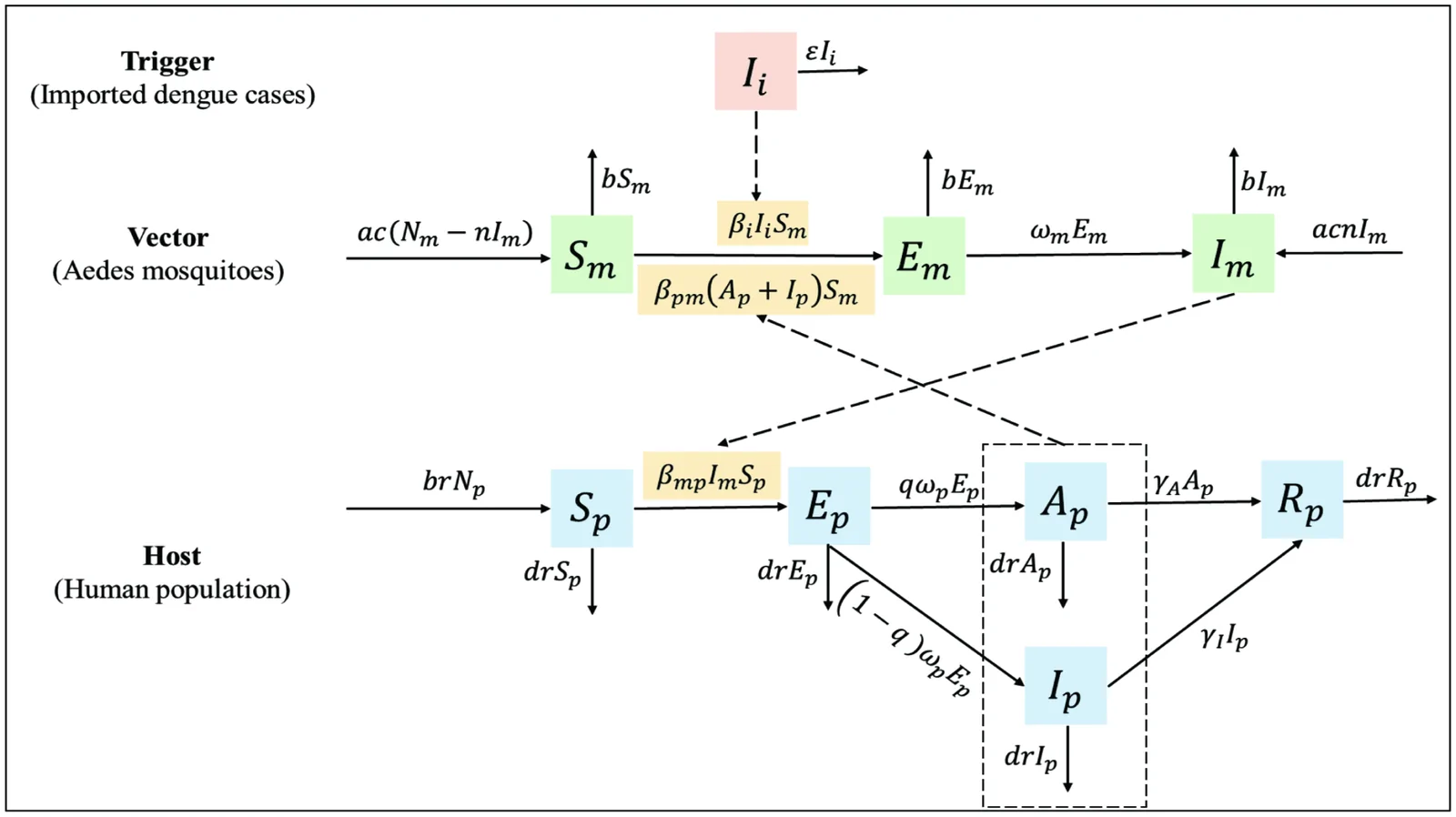
Flowchart of the mathematical model.

The assumptions of the model include:

(a) The transmission rate from imported dengue cases to local mosquitoes is denoted as $\beta_{i}$, and the transmission rates from local mosquitoes to susceptible individuals are denoted as $\beta_{mp}$, the transmission rates from infected individuals to susceptible mosquitoes are denoted as $\beta_{pm}$.
(b) We consider the birth, death, and vertical transmission of DENV within mosquitoes, where the birth rate, death rate, and vertical transmission ratio of mosquitoes are denoted as a, b, and n, respectively.
(c) Seasonality’s impact on transmission is modeled using a trigonometric function c, the formula to compute c is outlined below:

$$\begin{array}{c} c=cos\left[\frac{2\pi \left(t-\tau \right)}{T}\right] \end{array}$$

Where τ denotes the simulation delay of the initial time in the entire season, and T represents the duration of the season.

(d) We consider the birth and death rates within the human population denoted as $b_{r}$ and ${\;d}_{r}$.
(e) There exists a proportion of asymptomatic individuals among the infected human population, denoted as q.
(f) The incubation/latent period is indicated as $\frac{\text{1}}{\omega_{p}}$ and $\frac{\text{1}}{\omega_{m}}$ in the human and mosquito population, respectively.
(g) The recovery rate for infectious individuals is $\gamma_{I}\;$and for asymptomatic individuals is $\gamma_{A}$. In addition, the recovery rate for imported cases is denoted by ε.

The ordinary differential equation of the model is presented as follows:


$$\begin{array}{c} \begin{array}{c} \frac{dI_{i}}{dt}=-\varepsilon I_{i} \end{array} \end{array}$$ (1)

$$\begin{array}{c} \begin{array}{c} \frac{dS_{m}}{dt}=ac\left(N_{m}-nI_{m}\right)-\beta_{i}I_{i}S_{m}-\beta_{pm}\left(A_{p}+I_{p}\right)S_{m}-bS_{m} \end{array} \end{array}$$ (2)

$$\begin{array}{c} \begin{array}{c} \frac{dE_{m}}{dt}=\beta_{i}I_{i}S_{m}+\beta_{pm}\left(A_{p}+I_{p}\right)S_{m}-\omega_{m}E_{m}-bE_{m} \end{array} \end{array}$$ (3)

$$\begin{array}{c} \begin{array}{c} \frac{dI_{m}}{dt}=\omega_{m}E_{m}+\;acnI_{m}-bI_{m} \end{array} \end{array}$$ (4)

$$\begin{array}{c} \begin{array}{c} \frac{dS_{p}}{dt}=b_{r}N_{p}-\beta_{mp}I_{m}S_{p}\;-{\;d}_{r}S_{p} \end{array} \end{array}$$ (5)

$$\begin{array}{c} \begin{array}{c} \frac{dE_{p}}{dt}=\beta_{mp}I_{m}S_{p}-{\;\omega }_{p}E_{p}\;{-\;d}_{r}E_{p} \end{array} \end{array}$$ (6)

$$\begin{array}{c} \begin{array}{c} \frac{dI_{p}}{dt}=(1-q{\;)\omega }_{p}E_{p}\;-\;\gamma_{I}I_{p}{-\;d}_{r}I_{p} \end{array} \end{array}$$ (7)

$$\begin{array}{c} \begin{array}{c} \frac{dA_{p}}{dt}=q\omega_{p}E_{p}\;-\;\gamma_{A}A_{p}{-\;d}_{r}A_{p} \end{array} \end{array}$$ (8)

$$\begin{array}{c} \begin{array}{c} \frac{dR_{p}}{dt}=\gamma_{A}A_{p}+\;\gamma_{I}I_{p}{-\;d}_{r}R_{p} \end{array} \end{array}$$ (9)

$$\begin{array}{c} \begin{array}{c} N_{m}=S_{m}+E_{m}+I_{m} \end{array} \end{array}$$ (10)

$$\begin{array}{c} \begin{array}{c} N_{p}=S_{p}+E_{p}+I_{p}\;+A_{p}\;+R_{p} \end{array} \end{array}$$ (11)

There are 11 variables and 17 parameters. The summary of the variables and parameters is provided in Table 1.

**Table 1:** Variables and parameter definitions and values of the model.

Variables/ Parameters	Description	Unit	Value	Range	Sources
$I_{i}$	Imported infected individuals	Individuals	–	≥0	Actual data
$S_{m}$	Susceptible mosquitoes	Individuals	–	≥0	Modeling
$E_{m}$	Exposed mosquitoes	Individuals	–	≥0	Modeling
$I_{m}$	Infectious mosquitoes	Individuals	–	≥0	Modeling
$S_{p}$	Susceptible individuals	Individuals	–	≥0	Modeling
$E_{p}$	Exposed individuals	Individuals	–	≥0	Modeling
$I_{p}$	Infectious individuals	Individuals	–	≥0	Curve fitting/ Modeling
$A_{p}$	Asymptomatic individuals	Individuals	–	≥0	Modeling
$R_{p}$	Recovered/removed individuals	Individuals	–	≥0	Modeling
$N_{m}$	Total mosquito population size	Individuals	–	≥0	Actual data
$N_{p}$	Total human population size	Individuals	–	≥0	Actual data
$\beta_{i}$	Transmission rate from $I_{i}$ to$S_{m}$	1	–	≥0	Curve fitting
$\beta_{mp}$	Transmission rate from $I_{m}$ to$S_{p}$	1	–	≥0	Curve fitting
$\beta_{pm}$	Transmission rate from $A_{p}$ and $\;I_{p}$ to$S_{m}$	1	–	≥0	Curve fitting
a	The birth rate of mosquitoes	Day-1	0.071	0-0.071	[15,16]
b	The death rate of mosquitoes	Day-1	0.071	0-0.071	[15,16]
n	Vertical transmission ratio of the mosquitoes	1	0.1	0.0140–0.174	[17]
c	Seasonality parameter of the mosquitoes	1	–	0–1	Curve fitting
τ	Simulation delay of the initial time in the entire season	Day	–	≥0	Curve fitting
𝑇	Duration of the season	Day	–	≥0	Curve fitting
$b_{r}$	The birth rate of the human population	1	–	–	Actual data
$d_{r}$	The death rate of the human population	1	–	–	Actual data
q	Proportion of human asymptomatic infection	1	0.688	0-1	[18]
$\omega_{m}$	The incubation rate of mosquitoes	Day-1	0.100	0.083 - 0.125	[15]
$\omega_{p}$	The incubation rate of the human population	Day-1	0.167	0.125 -0.250	[19]
$\gamma_{I}$	The recovery rate of infectious individuals	Day-1	0.143	0.071 - 0.333	[15,16]
$\gamma_{A}$	The recovery rate of asymptomatic individuals	Day-1	0.143	0.071 - 0.333	[15,16]
ε	The recovery rate of imported cases	Day-1	–	0.071 - 0.333	Curve fitting

The model parameters were fitted to the cumulative number of local cases using the least squares method. Model fit was evaluated using the following criteria: R-squared (*R*^*2*^), Root Mean Square Error (RMSE), and Mean Absolute Error (MAE). The basic reproduction number (*R*_*0*_) quantifies dengue transmissibility, derived using the next-generation method [20] (see Supplementary text 3 in S3 Text for details).

To address uncertainty in parameter estimates, we performed four complementary analyses. First, we conducted a sensitivity analysis by stratifying parameters into 1000 values within the range from the reference; the mean and standard deviation (SD) of the simulated cases were then computed using our previous methodology [9,21]. Second, we assessed fitting stability using bounded multi-start optimization, in which the model was repeatedly fitted across 100 stratifying values per parameter within the range; we then summarized the minimum sum of squared errors (SSE) and the near-optimal ratio (the proportion of solutions within 5%). Third, by fixing each parameter at 1,000 points spanning the estimated value ± 3 SD and re-fitting the remaining estimable parameters, support regions were defined using a likelihood-ratio-type criterion at the 95% confidence level based on changes in the objective function. Fourth, we propagated uncertainty in multiple parameters using 500 rounds of Latin hypercube sampling (LHS), followed by per-sample re-fitting. Parameter bounds and grid domains were defined from Table 1. We calculated the mean fitted curve, the 95% confidence interval (95% CI), and coverage, defined as the proportion of observed data points that fell within the simulated 95% CI.

To assess the robustness of *R*_*0*_, we conducted a sensitivity analysis that mirrored the model-fitting simulations. Each key parameter was varied across 1,000 stratified values spanning its reference range, while all other parameters were held at their baseline values. *R*_*0*_ was then recalculated using the same next-generation matrix framework, and its mean and SD were summarized. We further propagated multivariate parameter uncertainty through 10,000 rounds of LHS, followed by refitting for each sample, and again recalculated *R*_*0*_. To quantify the contribution of individual parameters to the variance in *R*_*0*_, we calculated the first-order sensitivity index (S1) and the total-effect index (ST); higher S1 and ST values indicate that uncertainty in those parameters contributes most strongly to the overall uncertainty in *R*_*0*_.

### Statistical analyses

Data management was performed in Excel 2019, and data cleaning and analysis were conducted in Python 3.9 (Jupyter Notebook 7.3.2). Flowcharts were created using PowerPoint 2019. Continuous variables are reported as mean (SD) or median (interquartile range, IQR), according to the normality test results. Categorical variables are presented as counts and proportions. Statistical tests were selected according to variable type, number of groups, normality, and homogeneity of variance. Incidence rates were compared using Poisson regression with the corresponding population denominator entered as an offset term. Bonferroni correction was applied for multiple comparisons. A two-sided p-value < 0.05 was considered statistically significant.

## Results

### Epidemiological characteristics of dengue in Ningbo City 2017–2024

#### Epidemiological characteristics of dengue cases.

From 2017 to 2024, Ningbo City reported 266 dengue cases and no deaths. Among all cases, 27.44% (73/266) were local, 42.11% (112/266) were overseas imported cases, and 30.45% (81/266) were domestic imported cases. In Phase I, most cases were overseas imported (51.19%, 86/168) or local (43.45%, 73/168). No cases were reported in Phase II. In Phase III, most were domestically imported (73.47%, 72/98), and no local cases were identified (Fig 3A).

**Fig 3:**
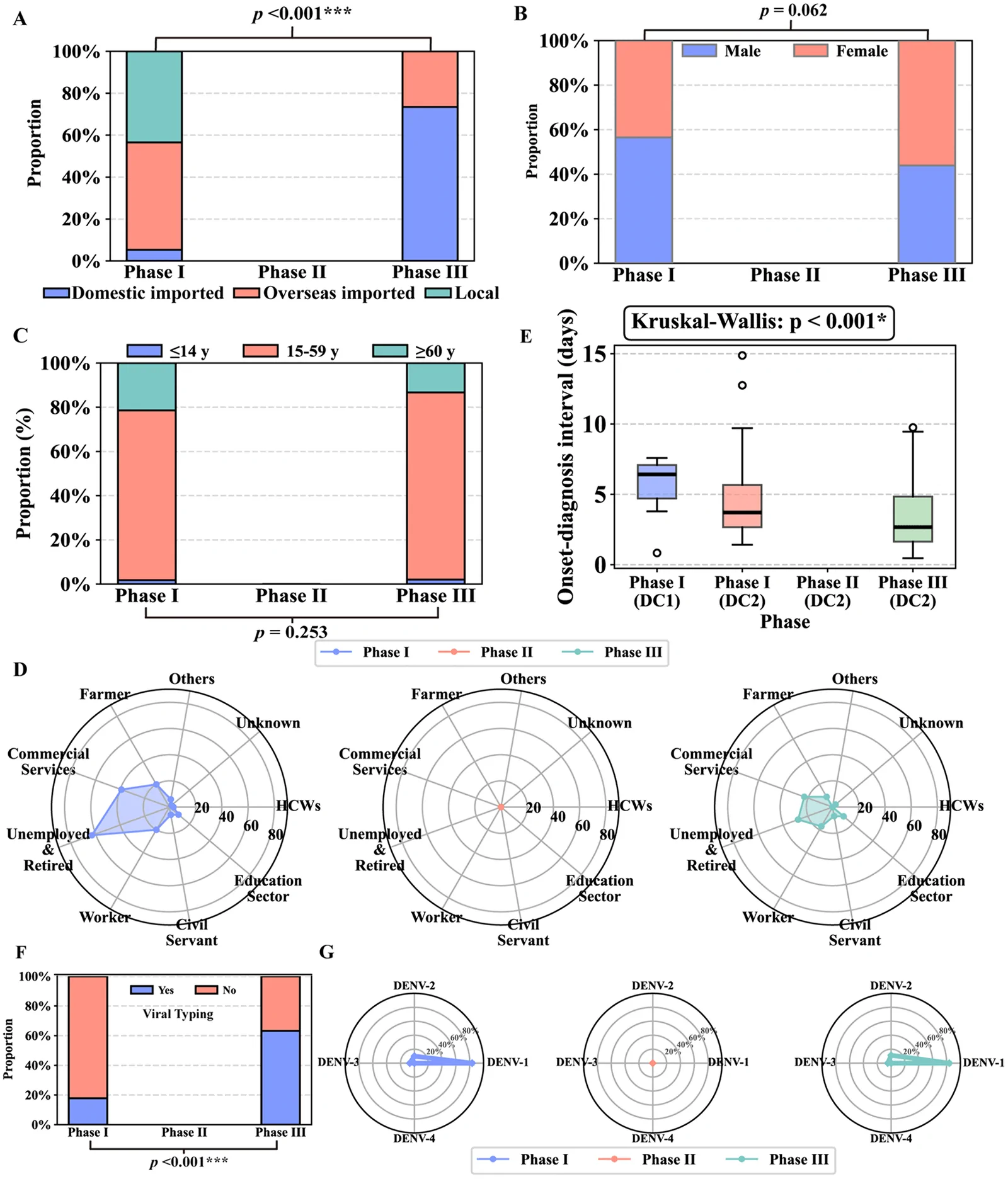
Comparison of the Demographic and Epidemiological Characteristics of Dengue Cases in Ningbo, 2017–2024. (A) Comparison of Dengue Cases from Multiple Data Sources in Ningbo, 2017–2024. (B) Gender distribution of dengue cases in Ningbo, 2017-2024. (C) Age distribution of dengue cases in Ningbo, 2017-2024. “y” in the legend denotes age (in years). (D) Occupational distribution of dengue cases in Ningbo, 2017-2024. HCWs, health care workers. (E) The onset-to-diagnosis interval distribution of dengue cases in Ningbo, 2017-2024. DC1, Diagnostic Criteria 1 (WS 216-2008); DC2, Diagnostic Criteria 2 (WS 216-2018). (F) Distribution of Viral Typing Rates Among Dengue Cases in Ningbo, 2017–2024. (G) The DENV distribution of dengue cases in Ningbo, 2017-2024. DENV, dengue virus.

Male cases (51.88%, 138/266) slightly outnumbered female cases (48.12%, 128/266), but the difference was not statistically significant (*p* = 0.435). The median age was 38.5 years (IQR = 31.25-54.00), and most cases occurred in the 15–59 age group (79.70%, 212/266) (Figs 3B–3C, S1 Fig). Age and sex composition did not differ significantly across different phases (*p* > 0.05) (Fig 3B–3C). The most common occupational groups were unemployed/retired (34.21%, 91/266) and commercial services (23.31%, 62/266), with no significant phase-specific difference in occupational distribution (*p* = 0.081) (Fig 3D).

The median onset-to-diagnosis interval across all cases was 3.67 days (IQR = 1.96-5.67). In Phase I, median onset-to-diagnosis intervals both before (median 6.42, IQR = 4.70-7.08) and after (median 3.7, IQR = 2.67-5.67) the diagnostic-criteria update were significantly longer than in Phase III (median 2.67, IQR = 1.63-4.84). (Figs 3E, S2, S1 Table). Viral typing results were available for 34.59% (92/266) of cases. The typing rate was significantly higher in Phase III (63.27%, 62/98) than in Phase I (17.57%, 30/168) (Fig 3F). Among typed cases, 83.70% (77/92) were DENV-1, and no DENV-4 was detected. Pathogen composition did not differ significantly across phases (*p* = 1.000) (Fig 3G).

In Phase I, dengue was sporadic, and no local cases were reported in 2017. The local outbreak emerged in 2018 and 2019, with August peaks of 31 and 28 cases, respectively (Fig 4A). During this phase, most imported cases were overseas imported cases, primarily from Cambodia (37.89%, 36/95), Thailand (16.84%, 16/95), and Vietnam (10.53%, 10/95) (Figs 4B, S3). During Phase II, coinciding with COVID-19 PHSM implementation, no cases were reported (Figs 4A, S4). In Phase III, after PHSM relaxation, imported cases rebounded, but no local transmission occurred. Imported cases peaked in September and October of 2023–2024 (24 and 12 cases, respectively), and most were domestic imported cases, primarily from Xishuangbanna, Yunnan Province (41.84%, 41/98), and Guangzhou City, Guangdong Province (14.29%, 14/98) (Figs 4B, S5).

**Fig 4:**
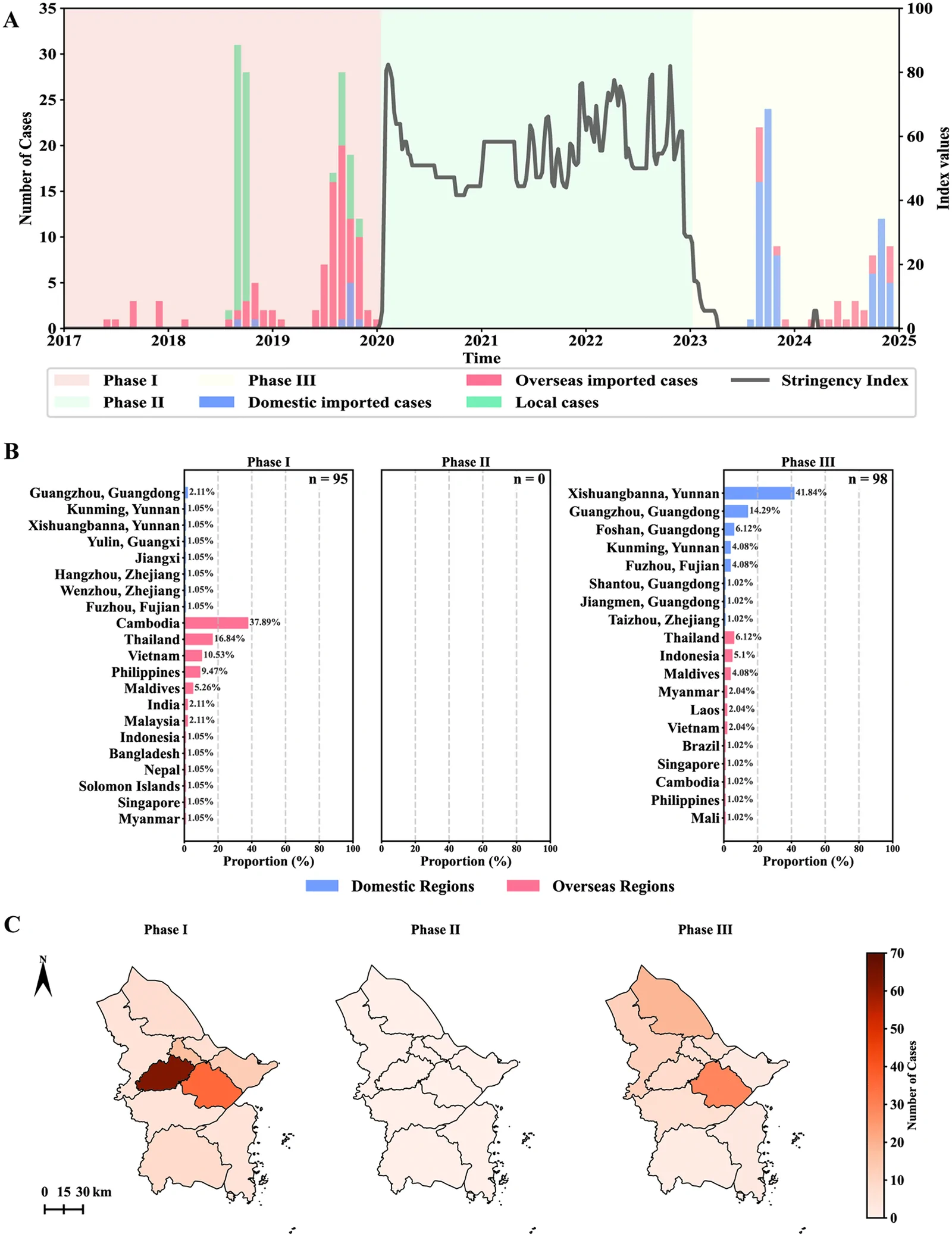
Spatiotemporal Distribution of Dengue Cases in Ningbo, 2017–2024. **(A)** Epidemic Curve of Dengue in Ningbo, 2017–2024. **(B)** Distribution of Source Regions for Imported Dengue Cases in Ningbo, 2017–2024. **(C)** Spatial Distribution of Dengue Cases in Ningbo, 2017–2024. The base map uses citywide and district administrative boundaries from the Database of Global Administrative Areas (GADM, https://gadm.org/data.html; license: CC BY 4.0, https://gadm.org/license.html).

Dengue cases were reported across all 10 administrative districts, with higher proportions in Haishu (27.07%, 72/266) and Yinzhou (24.44%, 65/266). The spatial distribution of dengue cases showed significant variation across phases (*p* < 0.001). During Phase I, cases were predominantly in Haishu (37.50%, 63/168) and Yinzhou (21.43%, 36/168). In Phase III, the concentration shifted to Yinzhou (29.59%, 29/98) and Cixi (19.39%, 19/98) (Fig 4C).

Dengue incidence in Ningbo during 2017–2024 was 2.82 per 100,000 persons. Incidence was higher in females than in males, but this difference was not significant (p = 0.904). In Phase I, incidence was higher in males, whereas in Phase III, incidence was higher in females; neither phase-specific sex difference was significant (p > 0.05). Incidence among persons aged 15 years or older was significantly higher than among those aged 14 years or younger, and this pattern was observed in Phases I and III. Pairwise comparisons showed no significant difference between the 15–59-year and 60-year-or-older groups. Across the three age groups, incidence differed significantly in Phase I; in Phase III, the 14-year-olds-or-younger and 60-year-olds-or-older groups did not differ significantly (Table 2, S6 Fig).

**Table 2:** The incidence rates of dengue by demographic group and study phase in Ningbo City from 2017 to 2024.

Type		Overall	p (Overall)	Phase I	p (Phase I)	Phase II	p (Phase II)	Phase III	p (Phase III)	p (Different phases)	p12	p13	p23
**All population**		2.82		1.86		0.00		1.01		<0.001*	<0.001*	<0.001*	<0.001*
**Sex**	**Male**	2.85	0.904	2.13	0.082	0.00	1.000	0.84	0.112	<0.001*	<0.001*	<0.001*	<0.001*
	**Female**	2.79		1.61		0.00		1.19		<0.001*	<0.001*	0.107	<0.001*
**Age group**	**≤14**	0.45	<0.001*	0.29	<0.001*	0.00	1.000	0.18	<0.001*	0.220			
	**15-59**	3.18		1.91		0.00		1.27		<0.001*	<0.001*	<0.001*	<0.001*
	**≥ 60**	3.01		3.01		0.00		0.63		<0.001*	<0.001*	<0.001*	0.003*
**District**	**HS**	6.90	<0.001*	6.23	<0.001*	0.00	1.000	0.84	0.025*	<0.001*	<0.001*	<0.001*	0.008*
	**JB**	4.93		3.77		0.00		1.36		<0.001*	<0.001*	0.030*	0.027*
	**YZ**	4.03		2.40		0.00		1.71		<0.001*	<0.001*	0.211	<0.001*
	**ZH**	2.76		1.44		0.00		1.33		0.029*	0.020*	1.000	0.026*
	**FH**	2.06		0.87		0.00		1.19		0.041*	0.071	0.816	0.025*
	**BL**	1.92		1.72		0.00		0.33		<0.001*	<0.001*	0.009*	0.273
	NH	1.56		1.29		0.00		0.28		0.002*	0.007*	0.065	0.485
	**CX**	1.47		0.45		0.00		1.01		<0.001*	0.011*	0.078	<0.001*
	**XS**	1.40		0.88		0.00		0.52		0.091			
	**YY**	1.37		0.41		0.00		0.95		0.002*	0.067	0.174	0.002*

HS, Haishu District; JB, Jiangbei District; YZ, Yinzhou District; ZH, Zhenhai District; FH, Fenghua District; BL, Beilun District; NH, Ninghai County; CX, Cixi City; XS, Xiangshan County; YY, Yuyao City; * indicates statistical significance at p < 0.05, *p12* = p-value of the pairwise comparison of incidence between phase I and between phase I and phase II, *p13* = p-value of the pairwise comparisons of incidence between phase I and phase III, *p23* = p-value of the pairwise comparisons of incidence between phase II and phase III.

Spatially, incidence was relatively high in Haishu and Jiangbei and relatively low in Cixi and Yuyao, with a similar pattern in Phase I. In Phase III, incidence was relatively high in Yinzhou and relatively low in Beilun and Ninghai (Table 2, S6 Fig). Incidence declined sharply from Phase I to Phase II and rebounded in Phase III. However, phase-specific incidence differences were not significant for the 14-year-olds or younger group or for Xiangshan County (Table 2).

### Epidemiological characteristics of vector surveillance

From 2017 to 2024, the Ningbo CDC surveyed 153,621 households and identified 25,454 positive containers; all positive containers contained *Aedes albopictus*. The mean BI was 16.07 (SD: 8.04). BI differed significantly across phases, with a higher mean in Phase I (22.49, SD: 7.78) than in Phase II (13.60, SD: 5.68) and Phase III (10.14, SD: 4.04) (*p* < 0.001) (Figs 5A, S7, Table 3). The BI also exhibited clear seasonality, with significantly higher mean values in summer than in other seasons across all three phases (*p* < 0.05) (Figs 5B, S8, S2 Table).

**Table 3:** Distribution of the Breteau Index in different regions at different phases and the results of their statistical tests.

District Name	M1 (SD)	M2 (SD)	M3 (SD)	*p*_*n1*_	*p*_*n2*_	*p*_*n3*_	*p*_*v*_	*p*	*p12*	*p13*	*p23*
Ningbo	22.49 (7.78)	13.60 (5.68)	10.14 (4.04)	0.588	0.548	0.327	0.072	<0.001*	<0.001*	<0.001*	0.207
Jiangbei	16.21 (10.31)	6.48 (3.28)	8.37 (4.58)	0.837	0.205	0.784	0.001*	<0.001*	<0.001*	0.005*	0.166
Beilun	10.96 (6.12)	14.53 (9.41)	9.34 (5.15)	0.390	0.759	0.812	0.058	0.189			
Zhenhai	13.64 (7.57)	7.55 (3.61)	8.72 (10.02)	0.246	0.143	0.362	0.992	0.003*	0.011*	0.011*	0.728
Yinzhou	15.94 (7.76)	12.79 (7.17)	11.80 (6.75)	0.460	0.523	0.858	0.688	0.200			
Xiangshan	24.68 (17.74)	13.96 (5.59)	10.71 (4.57)	0.274	0.248	0.180	0.015*	0.001*	0.036*	<0.001*	0.109
Ninghai	40.90 (23.70)	21.51 (13.26)	12.28 (5.01)	0.235	0.325	0.784	<0.001*	<0.001*	0.008*	<0.001*	0.063
Yuyao	16.97 (5.18)	14.94 (6.07)	11.02 (5.06)	0.819	0.891	0.960	0.774	0.006*	0.411	0.004*	0.078
Cixi	29.21 (16.30)	17.38 (8.44)	9.28 (4.40)	0.113	0.488	0.843	<0.001*	<0.001*	0.003*	<0.001*	<0.001*
Haishu	12.97 (9.76)	4.75 (4.73)	11.81 (7.46)	0.535	0.387	0.267	0.044*	<0.001*	0.002*	0.947	0.006*
Fenghua	NA	14.56 (6.41)	8.84 (3.70)	NA	0.901	0.716	0.035*	0.005*			

*p < 0.05. M1, Mean value of the Breteau Index for Phase I; M2, Mean value of the Breteau Index for Phase II; M3, Mean value of the Breteau Index for Phase III; SD, standard deviation; *p*_*n1*_, The p-value from the normality test in Phase I; *p*_*n2*_, The p-value from the normality test in Phase II; *p*_*n3*_, The p-value from the normality test in Phase III; *p*_*v*_, *p-*value from Levene’s test for homogeneity of variance*; p*, *p*-value of the central tendency of the Breteau Index across Phase I, Phase II, and Phase III*; p12*, *p*-value of the central tendency of the Breteau Index between Phase I and Phase II; *p13*, *p*-value of the central tendency of the Breteau Index between Phase I and Phase III; *p23*, *p*-value of the central tendency of the Breteau Index between Phase II and Phase III.

**Fig 5:**
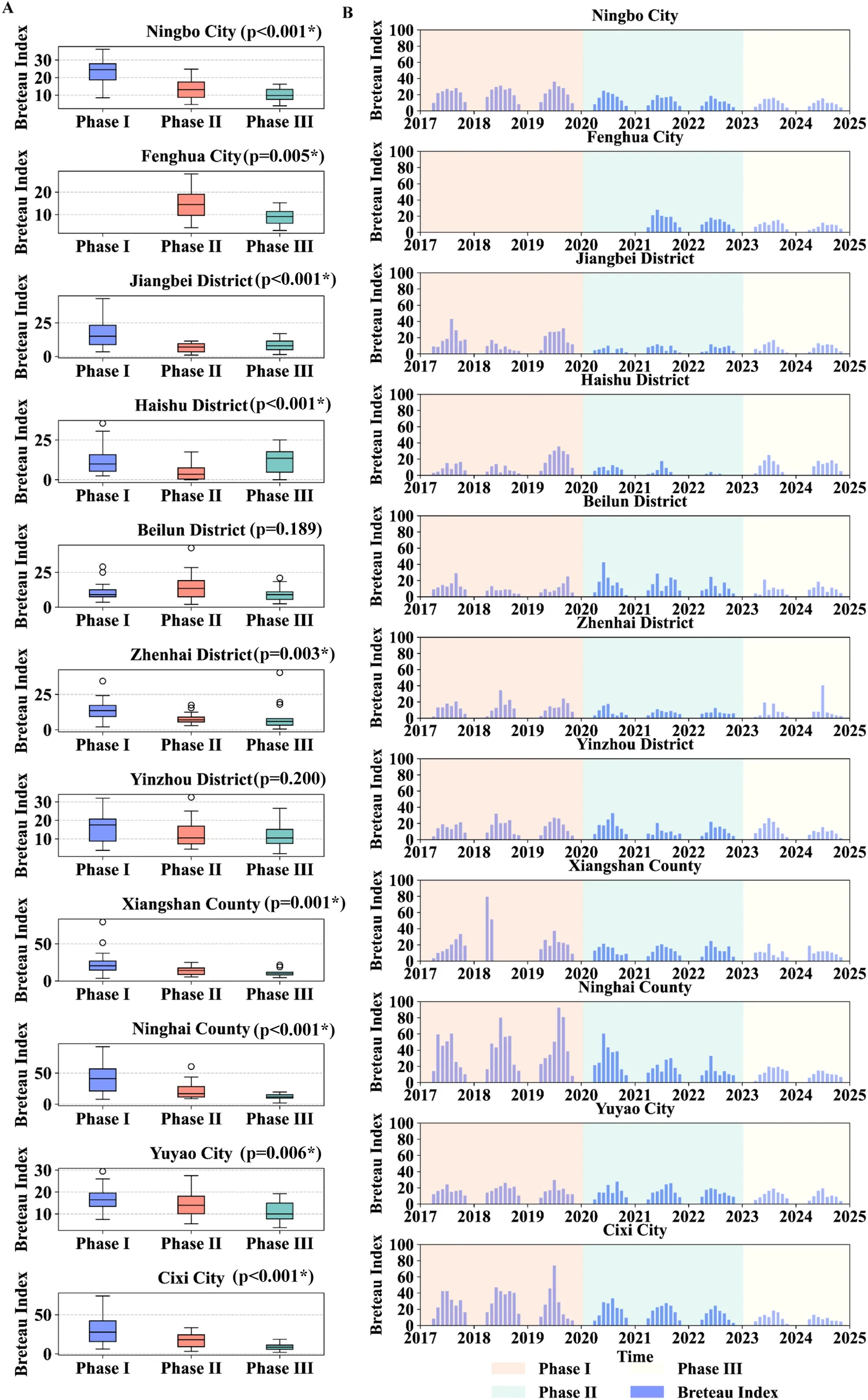
Mosquito vector density distribution in Ningbo, 2017-2024. **(A)** Comparison of Breteau Index values across administrative divisions of Ningbo during different surveillance phases. **(B)** Time-series trends of Breteau Index values in various administrative divisions of Ningbo.

By district, vector surveillance data were unavailable for Fenghua before 2020, and for Xiangshan through the end of 2018. Ninghai County had the highest mean BI (26.47, SD: 20.37), whereas Haishu had the lowest (9.60, SD: 8.41). In Jiangbei, Zhenhai, and Haishu, the mean BI in Phase II was lower than in Phases I and III; however, the difference was not significant in Jiangbei or Zhenhai, and the Phase I-III difference was not significant in Haishu. In Beilun, mean BI was highest, but phase differences were not significant. In Fenghua, mean BI was significantly higher in Phase II than in Phase III. In most remaining districts, BI followed the citywide pattern (Phase I > Phase II/III), although several comparisons were not significant, including Yinzhou, Yuyao and Haishu (*p* < 0.05). The mean BI in Phase II of Haishu was significantly lower than in Phase III. (Fig 5A, Table 3).

Seasonal patterns were broadly consistent across districts, with higher BI in summer than in other seasons. Notable exceptions included Beilun in Phase I, where the mean BI peaked in autumn, and in Xiangshan County in Phases I and III, where mean BI was higher in spring without statistical significance (Figs 5B, S8, S2 Table).

### Transmissibility of dengue in Ningbo City 2017–2024

Table 4 summarized fitted parameter values and model performance metrics for each time segment. Model fit was strong (R^2^ > 0.95), with an overall R^2^ of 0.995 (Table 4, Fig 6A). Sensitivity analysis indicated that the model was not sensitive to the parameters q. Our model is mildly sensitive to the parameter n,$\;\omega_{m}$, $\omega_{p}$, and $\gamma_{I}$, but more sensitive to a, b, and $\gamma_{A}$ (Fig 6B). The minimum SSE was 9.00 and was higher in 2018 than in other years; the near-optimal ratio was 0.94 and was lower in 2018 (S9A-S9B Fig). The 95% CI widths were larger for parameter ε than for $\beta_{i}$, $\beta_{mp}$ and $\beta_{pm}$ (1.08 vs. < 0.001), and were higher in 2019, except for $\beta_{mp}$ (S9C Fig). The coverage was 0.91, but only 0.33 in 2019. Although the coverage in 2018 was 83.33%, the 95% CI was widest (S9D Fig).

**Table 4:** Numerical results of evaluation indicators and fitted parameters for model fitting from 2017 to 2024.

Year	R^2^	RMSE	MAE	$\beta_{i}$	$\beta_{mp}$	$\beta_{pm}$	ε	T	τ
2017	1.00	0.00	0.00	0.00E + 00	0.00E + 00	0.00E + 00	4.29	12.00	7.15
2018	0.99	2.37	1.24	9.60E-09	2.14E-07	3.66E-04	9.82	12.00	3.34
2019	1.00	0.63	0.37	2.41E-08	1.56E-08	6.93E-03	9.15	12.00	3.57
2020	1.00	0.00	0.00	0.00E + 00	0.00E + 00	0.00E + 00	4.29	12.00	6.72
2021	1.00	0.00	0.00	0.00E + 00	0.00E + 00	0.00E + 00	4.29	12.00	6.87
2022	1.00	0.00	0.00	0.00E + 00	0.00E + 00	0.00E + 00	4.29	12.00	6.49
2023	1.00	0.00	0.00	0.00E + 00	0.00E + 00	0.00E + 00	4.29	12.00	6.96
2024	1.00	0.00	0.00	0.00E + 00	0.00E + 00	0.00E + 00	4.29	12.00	6.64

R^2^, Coefficient of Determination; RMSE, Root Mean Square Error; MAE, Mean Absolute Error.

**Fig 6:**
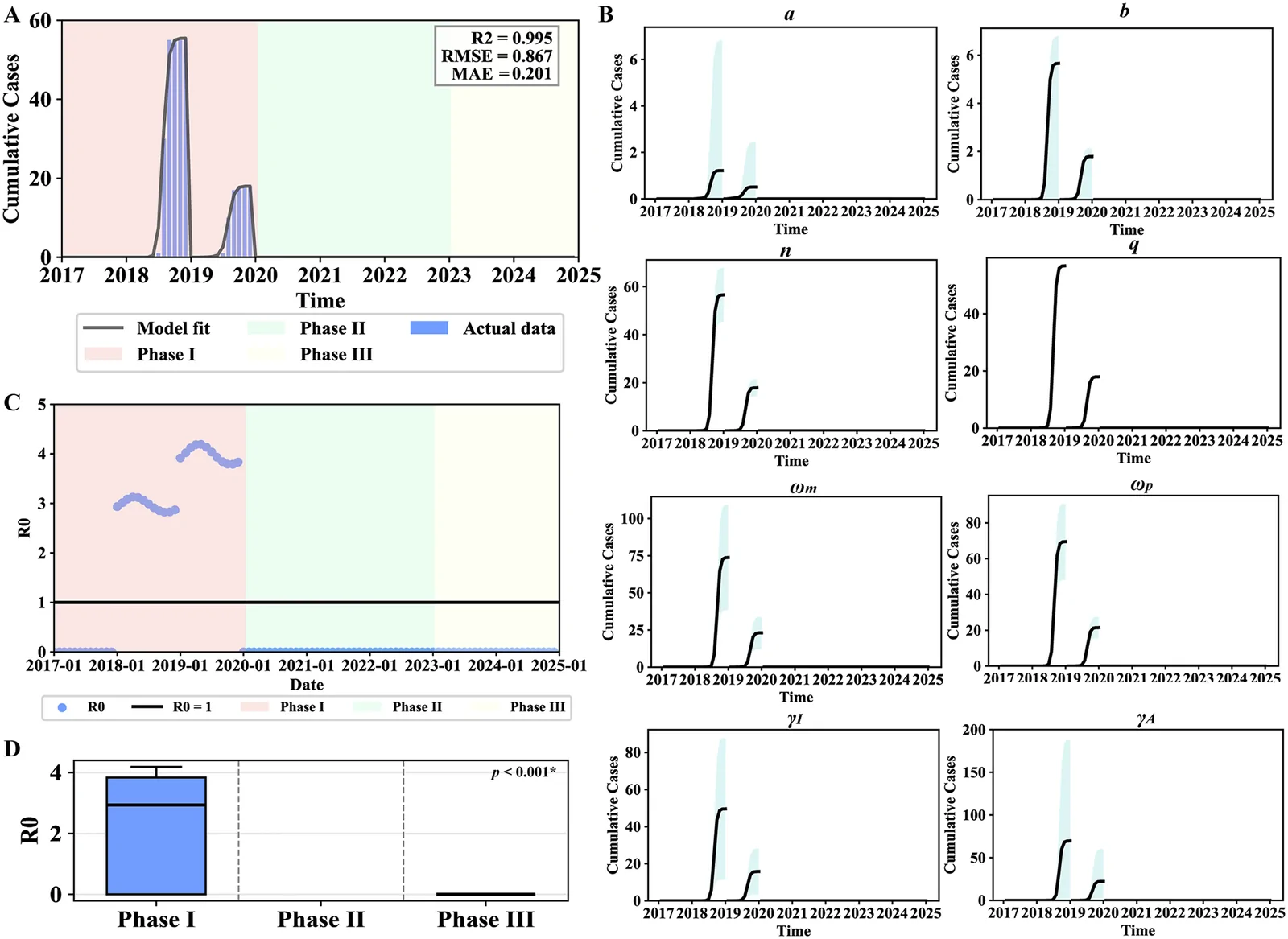
Mathematical Model Fitting and Transmissibility Estimation. **(A)** Model fit to the cumulative number of local dengue cases in Ningbo, 2017–2024. R^2^, Coefficient of Determination; RMSE, Root Mean Square Error; MAE, Mean Absolute Error. **(B)** Sensitivity analysis of key model parameters. **(C)** Estimated curve of the *R*_*0*_ for dengue in Ningbo, 2017–2024. **(D)** Distribution of the *R*_*0*_ of dengue in Ningbo across phases, 2017-2024. *R*_*0*_, Basic Reproduction Number.

The model-estimated median *R*_*0*_ for 2017–2024 in Ningbo was highest in the outbreak years (3.46, IQR = 2.98-3.95), whereas the years without local transmission yielded 0.00 (IQR = 0.00-0.00, *p* < 0.001). In 2018–2019, the mean *R*_*0*_ was 2.97 (95% CI = 2.90-3.04) and 3.98 (95% CI = 3.89- 4.08), respectively (Figs 6C–6D, S10). The median *R*_*0*_ over 2017–2024 was 0.00 (IQR = 0.00–0.71), reflecting the absence of local cases in most years. Across phases, median *R*_*0*_ was significantly higher in Phase I (2.94, IQR = 0.00-3.83) than in Phase III (0.00, IQR = 0.00-0.00) (*p* < 0.001) (Figs 6C–6D, S10).

Sensitivity analysis showed that the estimated *R*_*0*_ distribution was dominated by zero values in years without local transmission, which lowered the overall mean across the full study period. During outbreak years, *R*_*0*_ remained consistently above the epidemic threshold, ranging from 1.85 to 2,880.28 (mean: 7.00, SD: 10.09), whereas it remained 0 in non-outbreak years (S11A-S11I Fig). The uncertainty in *R*_*0*_ was dominated by parameter b (mean: 0.83; SD: 7.93; S1: 0.78; ST: 1.00); parameters a (mean: 0.86; SD: 1.51; S1: 2.77 × 10^-3^; ST: 0.16), n (mean: 0.87; SD:1.53; S1: 1.97 × 10^-3^; ST: 0.13), $\gamma_{A}$ (mean: 0.81; SD: 1.45; S1: 0.01; ST: 0.03), and $\gamma_{I}\;$(mean: 0.85; SD: 1.49; S1: 0.01; ST: 0.03) and q (mean: 0.87; SD: 1.53; S1: 1.72 × 10^-3^; ST: 0.01) had smaller but non-negligible effects; and $\omega_{m}$ (mean: 0.87; SD: 1.54; S1: 1.08 × 10^-4^; ST: 9.76 × 10^-5^) and $\omega_{p}$ (mean: 0.87; SD: 1.53; S1: 1.67 × 10^-7^; ST:1.94 × 10^-10^) contributed minimally, with near-zero sensitivity (S11A-S11J Fig).

## Discussion

In recent years, dengue incidence in Ningbo has increased, and its geographic distribution has expanded [22]. Based on our previous pre-pandemic analysis (2005–2018) [9], this study updates the epidemiological characteristics and transmission dynamics across three phases: before, during, and after the COVID-19 PHSM. These findings offer timely and relevant evidence for refining dengue control and prevention strategies.

No dengue-related deaths were recorded in Ningbo from 2017 to 2024, consistent with previous studies [22,23]. This pattern is compatible with Ningbo’s non-endemic status and with dengue well managed under China’s notifiable infectious disease framework [9,24]. The observed fatality profile differs from the global case-fatality estimate reported for 2024 [25]. We also found that most cases in Ningbo City during 2018–2024 were imported, whereas national reports for 2005–2023 indicated a larger proportion of locally acquired cases [24]. This discrepancy is epidemiologically plausible for a non-endemic setting in which local transmission is typically seeded by importation [9,23,24]. Local outbreaks in Ningbo (2018–2019) were limited and rapidly controlled compared with sustained outbreaks in high-risk provinces such as Guangdong and Yunnan [6,24].

Case composition in Ningbo exhibited distinct temporal variation. Imported and local cases co-occurred before the COVID-19 pandemic; no cases were reported during the strict PHSM implementation; and domestically imported cases reappeared after PHSM relaxation. This pattern is consistent with national observations [24]. Importantly, several factors may account for these changes: First, pre-pandemic cross-border mobility combined with rising global dengue activity may have increased importation risk [22,23]. Second, PHSM-related interventions—including international travel restrictions, community management, and environmental sanitation—may have reduced opportunities for dengue transmission in this non-endemic city [26,27]. Third, surveillance sensitivity for dengue may have changed because public health resources were prioritized for COVID-19 [23]. Fourth, the symptom overlap between dengue and COVID-19 symptoms may have contributed to under-ascertainment or diagnostic misclassification in some settings [28]. As these measures were PHSM relaxation, the resurged domestically imported cases may have been associated with renewed internal mobility linked to provinces with recurrent dengue activity (e.g., Yunnan, Guangdong) [24, 29–33]. Importantly, our dataset was designed to characterize temporal and spatial patterns, not to identify causal mechanisms. Accordingly, the above interpretations should be read as hypotheses rather than confirmed explanations. These mechanism-level hypotheses require further dedicated validation using mobility, diagnostic, and surveillance-performance data.

The median onset-to-diagnosis interval was 3.67 days, and this interval shortened across phases; it was also shorter than the interval reported for Zhejiang province (5–7 days) [23,24]. Several programmatic factors may have contributed, including repeated professional training, expanded use of rapid diagnostic testing, and implementation of a symptom-based supplemental surveillance system in Ningbo in 2023 [34]. Because these factors were not formally analysed in this study, their relative contributions should be interpreted as plausible but unquantified.

The viral typing rate increased across phases associated with repeated professional training. DENV-1 was the predominant serotype, consistent with previous findings [22,35,36]. This distribution may reflect importation links with areas where DENV-1 circulates widely, including the Pearl River Delta, the China-Myanmar border region in Yunnan, and parts of Southeast Asia [24,36–38].

Spatial differences in dengue cases were observed across phases: before COVID-19, cases were concentration in Haishu and Yinzhou; after PHSM relaxation, concentration was more evident in Yinzhou and Cixi. These changes may be related to differences in urban structure, population movement, and employment composition across districts [22]. Before the pandemic, Haishu—the urban and commercial core—and Yinzhou—an emerging urban area and international trade hub with the city’s international airport—were key destinations for international travellers and migrant workers, and most dengue infections were imported from abroad. After the PHSMs were relaxed, Yinzhou continued to grow through urban expansion, with significant population inflows into newly developed high-rise residential communities. Meanwhile, Cixi City attracted large numbers of domestic migrant workers due to its vast industrial zones, relatively low cost of living, and convenient transportation linking urban and rural areas. In the post-pandemic phase, as international trade and cross-border tourism had not fully recovered, most dengue cases were linked to domestic travel and internal labor mobility rather than international importation [34]. Nevertheless, our data do not directly test pathway-level drivers (e.g., tourism intensity, industrial structure, commuting networks), so these factors should be treated as hypothesis-generating explanations rather than established mechanisms.

The origin pattern of imported cases also shifted. Before the pandemic, overseas importation was more prominently associated with cross-border travellers and migrant workers to Southeast Asian countries such as Cambodia and Thailand [22]. After PHSM relaxation, domestic importation became dominant, with many importations linked to provinces reporting frequent dengue activity [24,34]. Although the dominant importation source changed, both periods indicate a strong relationship between mobility and importation risk in high-burden destinations. From a policy perspective, this supports coordinated prevention across health, transportation, customs, and culture-tourism sectors for travellers and returning workers from high-risk areas.

We found that the incidence of dengue fever in Ningbo City was higher than the national reported level. The overall trend was similar to the national report [24]. The incidence was higher before the COVID-19 pandemic and significantly decreased after the implementation of PHSM to deal with COVID-19; when these PHSM were relaxed, the incidence rebounded. The incidence in the ≥ 15-year-old age group was higher than that in the age group of ≤ 14 years old; this is consistent with what was found in other Asian countries that there was an excess of males among reported dengue cases ≥ 15 years of age; it suggests the importance of stratifying the reported incidence by gender and age, as using data that does not distinguish between genders and does not consider demographics may mask some of the true incidence situations [39]. After the relaxation of PHSM, the incidence rates rebounded in all regions, although there was no statistically significant difference among Beilun, Xiangshan County and Ninghai County. Especially in Fenghua, although the differences were not statistically significant, the incidence rate rebounded to a level higher than that before the COVID-19 pandemic. The spatial differences in incidence rates suggest that after the relaxation of PHSM, enhanced dengue prevention and control measures are needed, especially in areas like Fenghua, where the incidence rate has rebounded rapidly.

*Aedes albopictus* is the only vector across phases, consistent with a prior local study [9], and supporting the absence of an established *Aedes aegypti* population in Ningbo—unlike in parts of Yunnan, Hainan, and Guangdong, where *Aedes aegypti* is present [24]. Vector density gradually decreased after the COVID-19 pandemic and remained seasonally higher in summer, consistent with subtropical climatic suitability for *Aedes albopictus* [9,23]. The observed decline in vector density may have been influenced by emergency and routine vector-control activities [9,22].

Vector density also varied spatially: Most of the districts showed an overall decline, whereas district-specific fluctuations were observed (e.g., an increase-then-decrease pattern in Beilun and decrease-then-increase patterns in Jiangbei, Zhenhai). These differences may be associated with varying district characteristics. Beilun, an industrial zone, experienced reduced activity during COVID-19 prevention controls, leading to the accumulation of industrial waste containers such as tires, which provided favorable mosquito breeding habitats. Following the lifting of PHSM, resumed industrial operations and subsequent vector control activities helped reduce these breeding sites. In contrast, Jiangbei and Zhenhai are newly developed urban areas, while Haishu is an older urban district; all three have higher population densities. After pandemic controls ended, the resumption of population movement and a relaxation of prevention efforts contributed to a rebound in vector density in these areas. Therefore, district-tailored, continuously evaluated vector-control strategies remain warranted, particularly in areas with recent rebounds.

Mathematical models fitted the cumulative case curve from 2017 to 2024 with good performance metrics, indicating that the framework is useful for describing observed dynamics in Ningbo. Sensitivity analysis indicated more sensitivity to a, b, and $\gamma_{A}$. The model performed stably without local transmission. However, in years with local transmission, there were higher but acceptable fitting errors and wider intervals, suggesting that the stability and robustness were moderate. The wider interval width of the ε was much larger than the transmission rate, indicating that it has poor recognizability. Therefore, more field epidemiological investigation is needed for local empirical parameterization.

During periods with local transmission, the median *R*_*0*_ increased to 3.46, exceeding the epidemic threshold (>1) while remaining below some estimates from other subtropical settings [9,40]. These findings indicate the potential for short-term amplification when importation and favorable conditions coincide. Our sensitivity results provide a biologically coherent interpretation of transmission risk. The estimated transmissibility of dengue was low overall, consistent with the city’s non-endemic status [40]. In contrast, during outbreak years, *R*_*0*_ was consistently above the epidemic threshold, supporting the conclusion that short periods of secondary local transmission can occur when ecological and epidemiologic conditions align, even in a generally non-endemic city. The Sobol analysis indicates that uncertainty in *R*_*0*_ is driven primarily by mosquito survival, particularly the mosquito death rate, with smaller but still meaningful contributions from the mosquito birth rate and vertical transmission ratio. By comparison, other parameters contributed minimally. This pattern suggests that vector turnover, especially adult mosquito survival, is a key determinant of outbreak potential in Ningbo-like settings, whereas recruitment and vertical maintenance play secondary roles. These findings imply that control strategies aimed at reducing adult mosquito survival are likely to have the greatest effect on transmission potential, consistent with our previous findings and with observations from other non-endemic settings [9,41,42].

This study has several limitations. First, although the dengue case data were obtained from NNDRS, misclassification and underreporting cannot be excluded, particularly during periods when health-system resources were heavily allocated to the COVID-19 response. Especially in phase II, with no case reports, it affects the statistical test efficacy and the model estimation. Second, the completeness of mosquito surveillance data differed across districts, which may have affected vector-density estimates. Third, mosquito surveillance is conducted seasonally (April-November); extension to annual dynamics using trigonometric fitting may introduce model-based measurement error. Fourth, the scarcity of local-transmission observations, together with the reliance on literature-derived parameters rather than local field estimates, introduced additional uncertainty into the model simulations. As a result, the absolute magnitude of *R*_*0*_ should be interpreted cautiously, particularly for the outbreak years, because it is conditional on the assumed parameter values and model structure.

In conclusion, dengue in Ningbo remains importation-driven and non-endemic. Although Ningbo has improved diagnostic timeliness and reduced vector density, the rebound and persistent district-level heterogeneity indicate ongoing outbreak potential. A risk-stratified public health strategy that integrates mobility-informed surveillance, rapid diagnosis, and district-tailored vector control, with intensified summer operations and cross-sector coordination, is likely to provide the strongest protection against re-establishment of local transmission. For non-endemic port cities, this approach can reduce the risk of re-establishment.

## Supporting information

S1 Text An overview of the Dengue Surveillance System in Ningbo City. (DOCX)

S2 Text Vector Surveillance and Control in Ningbo City. (DOCX)

S3 Text Derivation of the next-generation matrix method for basic reproduction number (*R*_*0*_). (DOCX)

S1 Fig Age distribution of dengue cases in Ningbo City from 2017 to 2024. (TIF)

S2 Fig Distribution of the onset-to-diagnosis interval (ODI) for dengue cases in Ningbo City from 2017 to 2024. (TIF)

S1 Table Multiple comparisons of the adjusted p-values of the onset-to-diagnosis interval of dengue cases across phases in Ningbo, 2017–2024. (XLSX)

S3 Fig The Spatial Distribution of Imported Dengue Cases during Phase I in Ningbo City. (TIF)

S4 Fig The Spatial Distribution of Imported Dengue Cases during Phase II in Ningbo City. (TIF)

S5 Fig The Spatial Distribution of Imported Dengue Cases during Phase III in Ningbo City. (TIF)

S6 Fig The pairwise comparisons of incidence rates among different age groups and districts in Ningbo City from 2017 to 2024. (TIF)

S7 Fig Distribution of the Breteau Index (BI) in Ningbo City from 2017 to 2024. (TIF)

S8 Fig Distribution of the Breteau index in different districts of Ningbo City in different phases and seasons. (TIF)

S2 Table Comparison of Seasonal Distribution of the Breteau Index Across Different Phases and Regions. (XLSX)

S9 Fig Sensitivity analysis of multiple parameters of the mathematical model for dengue in Ningbo City. (TIF)

S10 Fig Distribution of the basic reproduction number (*R*_*0*_) in different districts of Ningbo City in different phases and years. (TIF)

S11 Fig Sensitivity analysis of the basic reproduction number (*R*_*0*_). (TIF)
