# Supplementary material for: Epidemiological characteristics and transmission dynamics of dengue in Ningbo City, China before, during, and after the COVID-19 pandemic interventions
Source: PLoS Negl Trop Dis. 2026 Oct 30;20(10):e0014813. doi: 10.1371/journal.pntd.0014813 (PMC13766620; doi:10.1371/journal.pntd.0014813)
Supplement: Supplementary file 1 [file pntd.0014813.s001.docx]

**Supplementary text 1. An overview of the Dengue Surveillance System in Ningbo City**

**1.1. Case definition**

All dengue cases are diagnosed based on the criteria established by the National Health Commission of the People's Republic of China, available at <https://www.chinacdc.cn/jkyj/crb2/yl/dgr/jswj_dgr/>. In 2017, the standard number we used was WS 216-2008, while since 2018, we have been using WS 216-2018. The case definitions and their changes for each diagnostic criterion are as follows:

Suspected case — a suspected case for WS 216-2008 is defined as either (a) compatible epidemiological exposure (travel to a dengue-endemic area within 14 days before illness onset, or residence/work in an area with recently reported dengue cases) combined with acute onset of fever, or (b) acute onset of fever accompanied by leukopenia or thrombocytopenia. The laboratory pathway requires either leukopenia or thrombocytopenia (not both) to meet the suspected-case definition.

The principal changes in WS 216-2018 are relaxation of the blood routine examination criterion from requiring both leukopenia and thrombocytopenia to requiring either condition, thereby increasing screening sensitivity.

Clinically diagnosed case — a clinically diagnosed case for WS 216-2008 is defined by two alternative routes. First, a suspected case arising from the epidemiological-exposure pathway that presents at least one typical clinical manifestation (for example, maculopapular rash or bleeding tendency) together with leukopenia or thrombocytopenia qualifies as clinically diagnosed. Second, any suspected case (meeting either epidemiologic or laboratory pathway) with detection of a positive dengue IgG or IgM.

The principal changes in WS 216-2018 are adding the titer of dengue IgG antibodies in the convalescent serum, which increased by 4 times or more compared to the acute phase and the detection of positive NS1 antigen of dengue virus within 5 days of onset as a criterion for the determination of the laboratory pathway.

Laboratory-diagnosed case— a laboratory-diagnosed case for WS 216-2008 is defined as confirmed if it meets the clinically-diagnosed definition and has at least one confirmatory laboratory result: virus isolation from acute specimens, detection of viral nucleic acid by RT-PCR or real-time RT-PCR, or a ≥4-fold rise in dengue IgG titer (or seroconversion) between acute and convalescent sera.

The principal changes in WS 216-2018 are expanded sources of laboratory-diagnosed cases, enabling direct confirmation of suspected cases with the same confirmatory laboratory evidence.

In addition, imported and local dengue cases were classified based on the site of infection through epidemiological investigations, according to the Dengue Surveillance Guidelines provided by the Chinese Center for Disease Control and Prevention (CCDC) available at <https://www.chinacdc.cn/jkyj/crb2/yl/dgr/jswj_dgr/>. These categories are mutually exclusive and collectively exhaustive. During the study period, this guideline remained unchanged.

(a) Overseas imported cases: Individuals who had travelled outside China to a country or region with documented dengue transmission within 14 days before symptom onset.
(b) Domestically imported cases: Individuals who had travelled outside their usual place of residence to another domestic area with known dengue transmission within 14 days before symptom onset.
(c) Local case: Individuals who had not travelled outside their usual place of residence, either internationally or domestically, within 14 days before symptom onset.

**1.2. Dengue Surveillance in China National Notifiable Infectious Disease Reporting Information System (NNDRS)**

The NNDRS is a national web-based surveillance system that collects data on 40 notifiable infectious diseases and other health-related topics in China. According to the national law on infectious disease prevention and treatment, notifiable infectious diseases are categorized as A, B, or C. Category A diseases must be reported to NNDRS within 2 hours, while categories B and C necessitate reporting to NNDRS within 24 hours.

Dengue is categorized as a class B notifiable infectious disease. Healthcare workers (HCWs) across all healthcare institutions in Ningbo City are obliged to report suspected, clinically diagnosed, or laboratory-confirmed dengue cases to the NNDRS within 24 hours. Subsequently, the Ningbo Center for Disease Control and Prevention (CDC) conducts epidemiological investigations, confirms diagnoses, and updates or supplements the information in the NNDRS based on the Guideline for Dengue prevention and control released by CCDC available at <https://www.chinacdc.cn/jkyj/crb2/yl/dgr/jswj_dgr/>.
