# Supplementary material for: Epidemiological characteristics and transmission dynamics of dengue in Ningbo City, China before, during, and after the COVID-19 pandemic interventions
Source: PLoS Negl Trop Dis. 2026 Oct 30;20(10):e0014813. doi: 10.1371/journal.pntd.0014813 (PMC13766620; doi:10.1371/journal.pntd.0014813)
Supplement: Supplementary file 2 [file pntd.0014813.s002.docx]

**Supplementary text 2. Vector Surveillance and Control in Ningbo City**

China categorizes provinces based on the severity of historical dengue outbreaks and the distribution of *Aedes* mosquitoes. Class I areas are provinces where dengue outbreaks have been frequent in recent years. Class II areas are regions with recent local cases or a higher risk of outbreaks due to the presence of *Aedes* mosquitoes. Class III areas have reported imported cases in recent years, indicating a risk of dengue outbreaks associated with *Aedes* mosquito presence.

Ningbo City is a Class I area for mosquito population surveillance. The city conducts vector surveillance and control in alignment with the guidelines issued by the Chinese Center for Disease Control and Prevention (China CDC) available at <https://www.chinacdc.cn/jkzt/crb/zl/dgr/jszl_2235/>. Surveillance is conducted annually from April to November, with two fixed and two mobile surveillance points established in each county or district. Each surveillance point inspects residential areas in four communities or villages, covering various geographical directions, with at least 100 households surveyed for small water containers and larvae breeding inside and outside structures. Positive mosquito larvae samples are identified for species classification either on-site or in labs through rearing until adulthood. To prevent any bias in calculating mosquito density, subsequent surveys are conducted in different households. A household is defined as a family unit, two rooms in shared dormitories, offices, hotels, or every 30 square meters in markets, nurseries, outdoor spaces, indoor public areas, and similar locales.

The Breteau Index (BI) is utilized for assessing *Aedes* mosquito density during surveillance. BI is calculated using the following formula:

$$BI = \frac{n_{p}}{N} *100$$

where $n_{p}$ is the number of positive water containers of *Aedes* mosquitoes, and $N$ is the number of households surveyed.

The measures implemented to control the mosquito population are as follows: Firstly, in coordination with Center for Disease Control and Prevention (CDC) professionals, the community is mobilized to eliminate stagnant water quantities in various high-risk locations (e.g., flowerpots, discarded tires, water tanks). Secondly, CDC employs insecticides for the swift eradication of adult mosquitoes. Outdoor mosquito management primarily entails ultra-low-volume spraying, complemented by residual spraying in mosquito habitats like livestock shelters and green areas. Conversely, indoor mosquito control emphasizes residual spraying, with specific locations requiring ultra-low-volume spraying. Thirdly, utilizing a variety of promotional platforms (e.g., brochures, newspapers, messaging, TV, and the Internet), CDC conducts health education on mosquito prevention and control. Moreover, social mobilization is undertaken to drive the Patriotic Health Campaign (PHC), motivating the population to actively participate in mosquito prevention and control measures. Last but not least, in surveillance regions where dengue fever cases are identified, and the BI exceeds 5, or the BI surpasses 20, the CDC instigates emergency interventions against the *Aedes* mosquito, encompassing eliminating stagnant water quantities in various high-risk locations and adult mosquitoes. Emergency measures cease when no new cases are documented within 25 days, and the BI drops below 5.
