# Supplementary material for: Epidemiological characteristics and transmission dynamics of dengue in Ningbo City, China before, during, and after the COVID-19 pandemic interventions
Source: PLoS Negl Trop Dis. 2026 Oct 30;20(10):e0014813. doi: 10.1371/journal.pntd.0014813 (PMC13766620; doi:10.1371/journal.pntd.0014813)
Supplement: Supplementary file 3 [file pntd.0014813.s003.docx]

**Supplementary text 3. Derivation of the next-generation matrix method for basic reproduction number (*R_0_*)**

We use the $R_{0}$ to estimate the transmissibility of dengue. The equation for $R_{0}$ was derived using the next-generation matrix (NGM) method as follows:

(1) Divide ($I_{i},S_{m},E_{m}, I_{m}, S_{p}, E_{p}, I_{p}, A_{p}, R_{p}$) into two categories: infected ($I_{i},E_{m}, I_{m},E_{p}, I_{p}, A_{p}$) and non-infected ($S_{m}, S_{p},R_{p}$).

(2) Divide the derivative of the infected category into two parts: $F$ represents the rates of new infections, $V$ represents the rates of transition between ($I_{i},E_{m}, I_{m},E_{p}, I_{p}, A_{p}$).

$$F - V =\left[ \begin{matrix} 0 \\ \beta_{i}I_{i}S_{m}+\beta_{pm}\left( A_{p}+I_{p} \right)S_{m} \\ \begin{matrix} 0 \\ \beta_{mp}I_{m}S_{p} \\ \begin{matrix} 0 \\ 0 \end{matrix} \end{matrix} \end{matrix} \right]- \left[ \begin{matrix} \varepsilon I_{i} \\ \omega_{m}E_{m}+bE_{m} \\ \begin{matrix} {-\omega}_{m}E_{m}- acnI_{m}+bI_{m} \\ \omega_{p}E_{p} {+ d}_{r}E_{p} \\ \begin{matrix} -(1-q{)\omega}_{p}E_{p} + \gamma_{I}I_{p}{+ d}_{r}I_{p} \\ - q\omega_{p}E_{p} + \gamma_{A}A_{p}{+ d}_{r}A_{p} \end{matrix} \end{matrix} \end{matrix} \right]$$

(3) Take derivatives of the vector-valued functions$F$ and $V$ to ($I_{i},E_{m}, I_{m},E_{p}, I_{p}, A_{p}$) to obtain the Jacobian matrices $JF$ and $JV$:

$JF =\left( \begin{matrix} 0 \\ S_{m}\beta_{i} \\ \begin{matrix} 0 \\ 0 \\ \begin{matrix} 0 \\ 0 \end{matrix} \end{matrix} \end{matrix}\begin{matrix} 0 \\ 0 \\ \begin{matrix} 0 \\ 0 \\ \begin{matrix} 0 \\ 0 \end{matrix} \end{matrix} \end{matrix}\begin{matrix} 0 \\ 0 \\ \begin{matrix} 0 \\ S_{p}\beta_{mp} \\ \begin{matrix} 0 \\ 0 \end{matrix} \end{matrix} \end{matrix}\begin{matrix} 0 \\ 0 \\ \begin{matrix} 0 \\ 0 \\ \begin{matrix} 0 \\ 0 \end{matrix} \end{matrix} \end{matrix}\begin{matrix} 0 \\ S_{m}\beta_{pm} \\ \begin{matrix} 0 \\ 0 \\ \begin{matrix} 0 \\ 0 \end{matrix} \end{matrix} \end{matrix}\begin{matrix} 0 \\ S_{m}\beta_{pm} \\ \begin{matrix} 0 \\ 0 \\ \begin{matrix} 0 \\ 0 \end{matrix} \end{matrix} \end{matrix} \right)$

$$JV = \left( \begin{matrix} \varepsilon\\ 0 \\ \begin{matrix} 0 \\ 0 \\ \begin{matrix} 0 \\ 0 \end{matrix} \end{matrix} \end{matrix}\begin{matrix} 0 \\ b+\omega_{m} \\ \begin{matrix} -\omega_{m} \\ 0 \\ \begin{matrix} 0 \\ 0 \end{matrix} \end{matrix} \end{matrix}\begin{matrix} 0 \\ 0 \\ \begin{matrix} b- acn \\ 0 \\ \begin{matrix} 0 \\ 0 \end{matrix} \end{matrix} \end{matrix}\begin{matrix} 0 \\ 0 \\ \begin{matrix} 0 \\ d_{r}\boldsymbol{+}\omega_{p} \\ \begin{matrix} -\omega_{p}\boldsymbol{(1-}q\boldsymbol{)} \\ -\omega_{p}q \end{matrix} \end{matrix} \end{matrix}\begin{matrix} 0 \\ 0 \\ \begin{matrix} 0 \\ 0 \\ \begin{matrix} d_{r}\boldsymbol{+}\gamma_{I} \\ 0 \end{matrix} \end{matrix} \end{matrix}\begin{matrix} 0 \\ 0 \\ \begin{matrix} 0 \\ 0 \\ \begin{matrix} 0 \\ d_{r}\boldsymbol{+}\gamma_{A} \end{matrix} \end{matrix} \end{matrix} \right)$$

(4) Construct the next generation matrix $G = FV^{-1}$. At the disease-free equilibrium ($I_{i},S_{m},E_{m}, I_{m}, S_{p}, E_{p}, I_{p}, A_{p}, R_{p}$) $= (0,{bN}_{m},0, 0, {brN}_{p}, 0,0, 0, 0)$*,*$R_{0}$ is the leading eigenvalue of $G:$

$$R_{0}= \sqrt{\frac{N_{m}N_{p}\beta_{mp} \beta_{pm} \omega_{m}\omega_{p}\boldsymbol{(}\gamma_{A}q\boldsymbol{-}d_{r}\boldsymbol{-}\gamma_{A}\boldsymbol{-}\gamma_{I}q\boldsymbol{)}}{(b+\omega_{m})(d_{r}\boldsymbol{+}\gamma_{A})(d_{r}\boldsymbol{+}\gamma_{I})(d_{r}\boldsymbol{+}\omega_{p})(acn - b)}}$$
